# Gaps in continuity of care: patients’ perceptions of the quality of care during labor ward handover in Mulago hospital, Uganda

**DOI:** 10.1186/s12913-015-0850-z

**Published:** 2015-05-06

**Authors:** Dan K Kaye, Annettee Nakimuli, Othman Kakaire, Michael O Osinde, Scovia N Mbalinda, Nelson Kakande

**Affiliations:** Department of Obstetrics and Gynecology, School of Medicine, College of Health Sciences, Makerere University, P.O. Box 7072, Kampala, Uganda; Department of Obstetrics and Gynecology, Jinja Regional Hospital, Jinja, Uganda; Department of Nursing, School of Health Sciences, College of Health Sciences, Makerere University, P.O. Box 7072, Kampala, Uganda; Clinical, Operations and Health Services Research Program, Joint Clinical Research Centre, P. O. Box 10005, Kampala, Uganda

## Abstract

**Background:**

Client satisfaction is a common outcome measure for quality of care and goal for quality improvement in healthcare. We assessed women’s perceptions of the structure, process and outcome of intrapartum care in Mulago hospital, specifically, labor ward duty shift handovers.

**Methods:**

Data was collected through 40 in-depth interviews conducted on two occasions: during the time of hospitalization and within 4–6 months after childbirth. Participants were women who delivered at the hospital, of whom some had life-threatening obstetric complications. Data was analyzed by thematic analysis.

**Results:**

Maternity duty handovers were associated with patient dissatisfaction, particularly the process of hand-over, the decision-making that follows handovers and failure of communication of information to patients and their caretakers. Consequently, duty handovers were perceived inadequate. They were described as gaps in the continuity of care, and contributed to poor quality of care, birth trauma and mothers’ dissatisfaction with the childbirth experience.

**Conclusion:**

The handover process and practices should be standardized using protocols and checklists. Health workers need training on handover practices, team work and communication skills (so as to improve patient-health provider and provider-provider interaction.

## Background

Ensuring high quality obstetric care is vital to reduction of maternal and neonatal mortality and morbidity in low income countries. Client satisfaction is one of the of commonly evaluated outcome measures for quality of obstetric care [1] and goal for improvement in healthcare [2-4]. Transitions of care occur throughout pregnancy and childbirth, and involve a variety of health professionals [5,6]. Duty shift handovers serve to ensure continuity of care between one group of healthcare providers and another [6-8]. Handovers involve change in numbers, seniority and experience of staff [5-8] and represent the formal transfer of responsibility and accountability [5-8] from one team of health workers to another. The handover has potential of creating discontinuity of care with regard to patient information or services [5-8]. Ensuring that the shared information is understood by oncoming teams is critical, as miscommunications may result in adverse outcomes for the healthcare teams and the patients [5-8].

Previous research at Mulago hospital reported poor quality of intaraptum care [9]. However, there is limited published research on the factors responsible for the poor quality of care. There is limited information on the professional conduct of health workers who provide intrapartum care, particularly on duty handover processes and practices in the hospital’s maternity unit. Our objective was to gain a deeper understanding the structure, process and outcome of intrapartum car during handovers through the clients’ perspective.

## Methods

This research was part of a post-doctoral research project of the first author (DKK) entitled *Evaluation and surveillance of the impact of maternal and neonatal near-miss morbidity on the health of mothers and infants in Jinja and Mualgo hospital.* The main objective of the project of this mixed-methods study is to assess preventable factors associated with maternal and neonatal near miss morbidity, from the perspective of patients and healthcare providers. The study was conducted in from June 1-October 30, 2013. The objective of this particular study was to gain a deeper understanding of mothers’ perspectives on quality of care (the structure, process and outcome of intrapartum care) particularly during duty handovers.

### Study setting

The study was conducted at Mulago hospital, Uganda’s national referral hospital and the teaching hospital for Makerere University. It has over 1500 beds, of which over 400 are maternity beds, and conducts over 35,000 deliveries per year. Health care is provided by a variety of health care professionals who include doctors (intern doctors, resident doctors who are on post-graduate trainng in Obstetrics and Gynecology or Family Medicine, Specialist Obstetricians of various ranks (registrars, consultants, senior consultants and university professors), nursing staff (midwives of various ranks), paramedical staff (laboratory staff, pharmacists, medical records staff and physiotherapists). In addition, there are medical students of Medicine, Nursing/Midwifery and other paramedical groups. The various staff are expected to formally change duty shifts regularly. The nursing/midwifery staff have a three-tier duty shift (day, evening and night) while the doctors have a two-tier duty shift changes (day and night) at 9.00 am and 5.00 pm every day.

### Participants

Participants were women admitted to Mulago hospital for delivery or due to complications of pregnancy or childbirth. Data was collected from through 40 in-depth interviews of 30 women, who were selected as follows: 10 had severe acute maternal morbidity, 10 had non-life-threatening complications, and 10 had uncomplicated deliveries with good maternal and newborn outcomes. Of the interviews, 26 were exit interviews conducted immediately after discharge (after some period of hospitalization for obstetric complications), 14 were follow-up interviews conducted 4–6 months after childbirth (conducted with women initially interviewed during hospitalization).

Participants were approached and requested to participate in the study during their hospitalization. Having agreed to participate, they were given assurance that the information given would be confidential, that their views would be anonymous. They were requested for permission to record the proceedings, after which they signed an informed consent. Their home addresses and telephone contacts were obtained so that they could be contacted where necessary for the postpartum interviews, which were conducted at their homes.

### Data collection

Data collection employed a phenomenological approach. Participants were selected by maximum variation sampling to represent a variety of age groups, education level, socio-economic status and relationship type. Issues explored were experiences of hospitalization and intrapartum care and perception of quality of duty handovers. Interviews lasted 40–60 minutes and were conducted in English and Luganda, a widely spoken local language, and proceedings were tape recorded.

### Data analysis

The data was analyzed by thematic analysis, using the framework by Donabedian [10]. This framework analyses the performance of practitioners, the contributions of patients and of the health care system; the social preferences of clients, and the causal linkages among the structural attributes of the settings in which care occurs, as well as the processes of care, and the outcomes of care. The transcripts were read independently by two members of the research team to identify patterns of words, phrases or statements across the dataset that described the phenomena (the essence of the phenomenon), to which codes were assigned. The two then compared the nature and type of codes generated. Agreement on which codes and categories were retained was by consensus. The codes were aggregated according to the identified meaningful patterns. A theme was identified as a consistent meaningful pattern of codes within the dataset that described or interpreted aspects of the phenomenon. Easy Text (EZ) software was used for data retrieval during analysis.

### Ethical considerations

Ethical approval to conduct the study was obtained from the Ethics and research committees of Mulago hospital (REC 310–2012), the School of Medicine, Makerere University College of Health Sciences (REC 2012–172) and Uganda National Council for Science and Technology Reference UNCST Rec UNCST 1381–2012). Permission to conduct the study was obtained from the department of Obstetrics and Gynaecology, Makerere University. All participants gave written informed consent to be interviewed and for proceedings to be recorded.

## Results

This study generated data about quality of care aspects (organization, structure, the process and outcomes) of intrapartum care specifically focusing on the duty handover period. Poorly organized, poorly-conducted and poorly supervised handover processes are a potential recipe for harm during intrapartum care. While this qualitative data may not indicate causality, it sheds light on the contextual factors of poor quality of care and associated severe maternal and perinatal morbidity. The study identified healthcare provider, health system factors and leadership and management-related factors related to handover processes which need to be improved in order to promote maternal and newborn survival and wellbeing during and after childbirth. Our objective was to describe handover as a phenomenon experienced or as perceived by mothers who sought intrapartum care. The objective was not to indicate whether poor handovers were associated with poor pregnancy or child birth outcomes, but to reveal the context and process by which adverse pregnancy outcomes may be related to poor handover practices and processes. The data may be utilized by clinicians, hospital managers, and policy makers for informing quality improvement programs for childbirth.

### Poor organization and poor conduct of handovers

All the women interviewed were aware that t health workers changed duty frequently, and did not expect to be looked after by the same individuals throughout their hospitalization. They were also aware of and expected that different teams of health workers would look after them during intrapartum care. The way in which handover (from one team of healthcare providers to another) was managed was believed to be key to women’s perception of quality of care and satisfaction with childbirth. Some of the participants reported having spent more than two days in the labor ward, and therefore witnessed several teams of health workers handing over to each other. All the participants indicated that there was no structured, formal or consistent approach to how handovers actually occurred or were conducted. The handovers ranged from a very brief exchange between teams of health workers, to a prolonged ward round where each and every admitted patient was reviewed. In either case, there was minimal involvement of the mothers in labor (or their attendants) in the handover process, as exemplified by one respondent:

*“I t was not done well. It was usually very brief. Often the doctors did not even look at you, let alone examine you. They only talked to themselves, often in a language you could not understand. There was no open communication. So you are left wondering what is happening, what they are talking about. Yet the doctors change all the time. They do not seem to be working as one healthcare team.”*

### Gaps in continuity of care during and after handovers

For some women, continuity of care was maintained because the new teams showed them more care and reassessed them with urgency after the handover process. In that respect, appearance of different people was a positive change, that positively impacted on the subsequent process of care.. The women described this as ‘signs of hope’, ‘reassuring’ or ‘relief’. Such women felt more satisfaction with the handover process. For other women, the new teams merely focused on finishing the handover round as soon as possible, without giving the mothers much attention or addressing their problems and needs. This often necessitated the on-coming teams to repeat the ward rounds, inevitably leading to inefficiencies and delays. Such women described the handover experience as being ‘abandoned’ by the previous teams after the hasty duty handovers. One woman describes this situation as very ‘frustrating’ or ‘uncertainty’: *The handover process varied greatly between individuals and between different teams, thus indicating problems in the organization and strudture of care and consequemnly, the process of care during and after handovers. The handover therefore created gaps in continuity of care*

*“There should be a better method of transfer of care. The new teams often went through the same questions that had been asked earlier without checking your file. This was and duplication of effort. Other times they just checked your file without re-assessing your situation or taking any action. At times doctors stop reviewing patients before they reach you.”*

The handover process often led to what women thought was indecision. Some women indicated that critical information is frequently not transmitted between health professionals, wrong decisions were taken or delays in receiving care were caused. Errors were related to omissions of vital content that is required for synthesis and rational judgment for assessment of the clinical condition of the patient, evaluation of results of investigations, critique of an ongoing management plan or assessment of the patient’s prognosis. This is exemplified by one aggrieved mother who developed a ruptured uterus after obstructed labor:

*“Some doctors make wrong diagnoses or make wrong decisions. And when one group comes to replace the one that has been treating you, they change the treatment, without asking you any questions or examining you. One team tells that you are for an operation, another team cancels the operation or tells you that nothing was written. Nobody asks for your opinion and rarely do they answer your questions during rounds.”*

### Traumatic experiences and negative outcomes of care related to handovers

The participants were aware that it was necessary for health workers to change duty in order to get some rest. Where they knew that they would be unavailable, (it was expected that) health workers would ask colleagues to cover their duties. However, most participants believed that duty shift change, duty transfers and poorly coordinated or poorly communicated health worker sign-offs triggered off several problems that affected quality of care or continuity of care. Such negative outcomes of care related to poorly organized or poorly conducted handovers included problems in decision-making for emergency care (wrong decisions could be made or there could be delays in decision-making). Other errors could be related to drug prescriptions (changing the route, timing or duration of treatment), poor interpretation of results of investigations (different teams could interpret the results differently) and poor evaluation of treatment or patient management plans (errors could be made regarding subsequent women’s care during patient follow-up). Where such errors occurred, participants reported negative or even traumatic experiences.

On their interaction with healthcare providers, some women described the health worker behaviour as ‘negligence’, ‘degrading’, ‘inhumane’ and ‘horrific.’ Even when they felt satisfied with the overall care received, some participants identified the duty shift handovers as partly responsible. One woman, who waited for one week for an elective caesarean section but had to undergo an emergency caesarean section, believed poor handover processes were partly responsible, exemplifies this view:

*“I’m very happy with the care I received while on the ward waiting for the operation. However, I am not happy that I had to go though labor pains as I was operated on by the night team as an emergency case, yet I was not supposed to go through labor. During the many hours of waiting, nobody explained to me what was going on.”*

For many women who developed and survived severe complications of childbirth, childbirth was a difficult period described as ‘trauma’, ‘degrading’, ‘terrible’, and ‘a period of suffering.’ Their description of the experience depicted anguish and grief. Women perceived childbirth negatively if medical intervention occurred, if the mode of delivery was not by natural birth or if they perceived negatively the treatment from healthcare professionals. This is exemplified by one woman who had a ruptured uterus following obstructed labor, and had a postpartum interview 4 months later:

*“I just keep thinking about it all the time. One of the hardest things ever was walking out of the hospital empty handed. I still feel the pain. They regularly checked on me, but nobody explained to me what happened.…At first the midwives said they were not aware of my case. I keep getting flashbacks all the time. I wonder how my baby looked like. I am still so upset. I can believe I will never get another child.”*

### Poor handovers as a feature of health system failures

Several mothers reported that health workers could not communicate to them about theirhealth status or the health status of their babies during handovers. . Neither did the healthcare providers seem concerned about the mothers’ anxiety. The mothers therefore felt left out in the decision-making at that critical time, when important decisions were being made. The participants were thus able to distinguish between human aspect healthcare and professional/technical skills. However, the consensus view was that these two aspects were essential and complementary. This is exemplified by one mother:

“*When the baby was born, they took her away and I could see frantic activities to revive the baby. They put the baby on oxygen briefly before whisking her away to the intensive care unit. They briefly showed me the sex of the baby. Yet for other babies, they would put them on top of the mothers’ abdomen. They even let mothers hold them or even kiss them. For me I never got back my baby until after 5 hours, after several inquiries to a new team of midwives. This really made me frightened. Later I had problems with breast feeding, so they had to return the baby to the nursery.”*

Many women needed to be communicated to about the health status of the baby, but often this was not done, particularly by new teams that took over work. This is illustrated by one mother who yearned for contact with her baby after premature childbirth:

*“They took him away without telling me what they were going to do or even getting my consent. The new team seemed unaware of what happened. For a whole day I was not allowed to touch him and that was really frustrating. They could not even let me take his photograph.”*

## Discussion

This was a phenomenological study that explored, using the Donabedian quality of care framework, the phenomenon of duty shift handovers and the perception of women on the quality of care received during and after the duty handover. Our results indicate that patients perceived duty handovers (shift change, duty transfers and health worker sign-offs) as triggers for several problems that affected quality of care, created gaps in continuity of care, and increased patient morbidity or mortality (from delayed or wrong decisions, inappropriate use of laboratory or diagnostic tests and failure to implement prior treatment plans). In addition, handovers often led to communication failure. The potential benefit of having a patient handled by fresh and less tired health workers (through changing duty shifts), might be offset by communication breakdown associated with sub-optimal handover and sign-out processes. Handover practices that lead to missed opportunities for appropriate care lead to poor quality of intrapartum obstetric care, with consequent maternal and perinatal morbidity and mortality. The findings reveal a link between inadequate communication among health workers during handovers and adverse consequences experienced by patients, and which are directly or indirectly attributable to health care providers. Staff-client/patient interaction, provision of information, involvement in decision-making, pain relief, positive birth environment and positive outcomes are related to satisfaction with the handover processes during intrapartum care. Satisfaction is a judgment formed by individuals as they reflect on their experiences, and is achieved when the patient/client’s perception of the services received is positive and/or meets their expectations [11].

Our findings suggest that duty shift handovers that are characterised by rushed, routine, and mechanical health worker-patient interactions are associated with patient dissatisfaction, are potentially harmful to women’s emotional wellbeing, and are perceived as an indicator of poor quality of intrapartum care. Duty shift handovers are characterised by inconsistent evaluation of the patients, reduced productivity and irrational decision making. Some health workers were noted ignore certain patients. Therefore, patient satisfaction surveys of the handover practices are critical to efforts on improvement of the quality of intrapartum care [12-14].

The reason for this is that transitions of care are critical to outcomes of intrapartum care, as inadequate sign-out and handover practices and routines have a significant toll on timely and efficient care. It is on handover ward rounds where clinical diagnosis (history and clinical examination) is revised, decisions regarding future investigations and treatment options are made, written and verbal communication on treatment plans are made to new teams. This may affect the subsequent care received by the patients. Unfortunately, after handovers, there might be a change in number of staff on duty, with fewer staff covering night and evening duty compared to day duty. Secondly, the night and evening duties are mainly covered by resident and intern doctors, under supervision of a specialist, who often is not available physically but is on call. Indeed, during and after duty shift changes, clinical teams that provide care to patients change in terms of speciality, seniority or number [5-8,15]. Thirdly, shift handovers are not satisfactorily performed if they do not focus on identifying and promptly addressing obstetric complications. Lastly, to enhance the safety and quality of these transitions of duty hand-over, communication is paramount to ensure formal transfer of responsibility and accountability between individuals or teams and teams of healthcare providers [15-18].

Therefore, handover represent transitions in care where or during which important decisions that ultimately influence outcome of patient care are made [19-24]. Sharing information and ensuring that this information is understood by oncoming staff is vital part of the handover process, as errors in handovers may contribute to adverse outcomes [19-24].

### Strength and limitations

For strengths, the study involved interviews of women whose experiences ranged from uncomplicated deliveries through non-life-threatening complications to maternal near miss morbidity. In addition, some of the women were interviewed both after their hospitalization and 4–6 months later (after hospitalization). Furthermore, the study generated data about quality of care aspects that need to be improved in order to promote maternal and newborn survival and wellbeing during and after childbirth. The data may be utilized by clinicians, hospital managers, and policy makers for informing quality improvement programs for childbirth. It is well established that clinical handover between healthcare providers is a high-risk activity for patient safety. In addition, in the existing literature, the importance of having in place robust handover practices and policies is extremely important. Several studies have shown that many hospitals do not have clearly defined handover policies and procedures and their practices fall short of the international recommendations. What the study adds on existing literature is the client perspective on the quality of care related to the handover processes and practices during intrapartum care. The study findings highlight the need to make clinical handovers a quality improvement priority for healthcare services, particularly during intrapartum care.

The limitations of this study are that it was cross-sectional and merely provides a snapshot of events. Secondly, the study is qualitative in nature, therefore cannot establish causality of adverse pregnancy outcomes attributable to the handover process. However, the study results highlight the context in which handover processes may be related to poor quality of care and subsequent adverse obstetric outcomes. In adition, there is no data on direct observation of the handover process, nor evaluation of the health workers’ perspective. Such perspectives would have explained or validated the data collected on patients’ perceptions. Thirdly, different hospitals differ in administrative policies, organizational structures, training protocols and considerations such as duration and organization of duty shifts. This is a challenge to generalizability of the recommendations to hospitals with different duty shift changes. Much as the study was conducted on patients from a single institution, the findings are applicable to many regional hospitals where duty shift staff changeovers occur.

## Conclusions

These results suggest that more should be done to improve quality of intrapartum care at Mulago hospital. There is need to explore how handovers can be improved such that the quality of care during handovers is improved and patients’ experiences of dissatisfaction are reduced. There is need to identify and implement standardized duty handover processes and practices so as to ensure acceptable patterns and routines during intrapartum care. Handover periods should allow shift overlap to permit adequate provider-provider and provider-patient interactions. Healthcare providers need to maintain patient confidentiality and privacy during the handover process.
